# Can Gut Microbiota and Lifestyle Help Us in the Handling of Anorexia Nervosa Patients?

**DOI:** 10.3390/microorganisms7020058

**Published:** 2019-02-22

**Authors:** Vanessa Mendez-Figueroa, Jose Miguel Biscaia, Rosa B. Mohedano, Ascension Blanco-Fernandez, Maria Bailen, Carlo Bressa, Mar Larrosa, Rocio Gonzalez-Soltero

**Affiliations:** Faculty of Biomedical and Health Sciences, Universidad Europea de Madrid, 28670 Villaviciosa de Odón, Spain; vanessamendez@hotmail.com (V.M.-F.); josemiguel.biscaia@universidadeuropea.es (J.M.B.); rosabelen.mohedano@universidadeuropea.es (R.B.M.); ascension.blanco@universidadeuropea.es (A.B.-F.); maria.bailen@universidadeuropea.es (M.B.); carlo.bressa@universidadeuropea.es (C.B.); mar.larrosa@universidadeuropea.es (M.L.)

**Keywords:** eating disorders, anorexia nervosa, gut microbiota, dysbiosis, gut–brain-axis

## Abstract

Gut microbiota is composed of different microorganisms that play an important role in the host. New research shows that bidirectional communications happen between intestinal microbiota and the brain, which is known as the gut–brain axis. This communication is significant and could have a negative or positive effect depending on the state of the gut microbiota. Anorexia nervosa (AN) is a mental illness associated with metabolic, immunologic, biochemical, sensory abnormalities, and extremely low body weight. Different studies have shown a dysbiosis in patients with AN. Due to the gut–brain axis, it was observed that some of the symptoms could be improved in these patients by boosting their gut microbiota. This paper highlights some evidence connecting the role of microbiota in the AN onset and disease progress. Finally, a proposal is done to include the microbiota analysis as part of the recovery protocol used to treat AN patients. When conducting clinical studies of gut microbiota in AN patients, dysbiosis is expected to be found. Then the prescription of a personalized treatment rich in prebiotics and probiotics could be proposed to reverse the dysbiosis.

## 1. Introduction

The intestine hosts a large community of microorganisms, called microbiota, which live in symbiosis with the host. Microbiota is composed of: archaea, protozoa, viruses and, above all, bacteria that exceed the number of human cells. The microbiota composition is like a fingerprint, unique and specific for each human [1]. The microbiota changes in number and type of species along the intestine, and its density and composition are affected by chemical, nutritional and immunological factors. For example, the small intestine contains elevated levels of acids and oxygen and the transit time is fast due to peristaltic movements. These factors limit bacterial growth, thus only those fast-growing bacteria will be able to adhere to the mucosal epithelium. In the colon, the environmental conditions are different, and therefore it can be inhabited by larger communities of bacteria, especially anaerobes. In fact, the majority of these bacterial communities are found precisely in the colon [2].

Anorexia nervosa (AN) is a mental illness that endangers the life of those who suffer from it. According to the Diagnostic and Statistical Manual of Mental Disorders (DSM-V) criteria, patients who maintain a diet restriction in relation to their energy needs, present a significantly lower weight than expected (according to their age, gender, development, and health status), or an intense fear for gaining weight are diagnosed with AN and can be classified as restrictive (self-induced vomiting, laxative abuse, diuretics or enemas during the last three months) or compulsive (binge-eating/ purging) [3]. Furthermore, AN is associated with metabolic, immunological, biochemical, sensory abnormalities, and an extremely low body weight [4,5,6]. Patients present alterations in the perception of their weight or their corporal silhouette, exaggeration of feeding importance or denial of the danger that the low weight implies. Weight recovery in patients with AN is essential for the prevention of somatic and psychological sequelae as osteoporosis, infertility or depression [7].

## 2. The Brain–Gut Axis and Possible Implications for Anorexia Nervosa

The Brain–Gut axis is the bidirectional communication system between the gut and the host brain that is regulated by neural, endocrine, and immunological systems and in which microbiota plays a key role [8,9]. The brain–microbiota communication is complex and carried out in several ways. One of these is the communication to the nervous system through the enteric nervous system (ENS). These connections coordinate and control the secretions, motility, mucosal transport and blood flow of the gastrointestinal tract that directly influence gut microbiota composition. The system works by means of motor neurons located in their ganglia, which act as effector cells of the gastrointestinal tract [10]. The ENS is connected to the central nervous system (CNS) by means of the vagus nerve, thus creating direct neurochemical signals from the gut microbiota to the brain [11], and from the nervous system to the gut microbiota [12].

**Figure 1:**
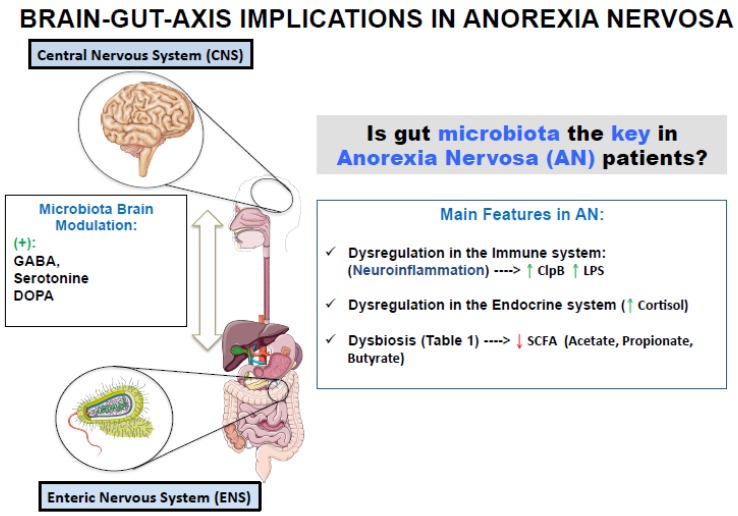
Microbiota as a recruiter of bidirectional communication between the brain and the gut and changes in microbiota composition observed in Anorexia Nervosa patients [15,17,19].

There is a plethora of molecules that connect the brain and the gut microbiota. In animal studies, it has been observed that the absence of microbiota provokes an abnormal development of the hypothalamic–pituitary–adrenal axis (HPA axis), that controls the release of cortisol which is the stress induced hormone [13]. The gut microbiota also impacts on endocrine glands that produce sexual and thyroid hormones and is implicated in the regulation of the peptides and hormones release, involved in the body mass energy balance and feeding behavior [14]. It has been proposed that certain microbiota microorganisms can induce an effect (positive or negative) on the host food habits and emotional behavior through the secretion of molecules [15]. On the other side, bacteria have receptors for these hormones, so they can communicate with the host’s brain [16]. For example, *Lactobacilli* and *Bifidobacteria* are capable of synthesizing the neurotransmitter gamma-aminobutyric acid (GABA) that reduces anxiety and stress, while *Escherichia*, *Bacillus*, and *Saccharomyces* produce norepinephrine [17]. Serotonin has been isolated from *Candida*, *Streptococcus*, *Escherichia*, and *Enterococcus*, and dopamine, the major disruptor of the CNS [18], is one of the final products of the metabolism of *Bacillus* and *Serratia* [19]. Moreover, the gut microbiota also produces butyrate acid that can exert numerous beneficial effects in brain [20], but also release the lipopolysaccharide (LPS) to the bloodstream that crosses the blood-brain barrier and can induce neuroinflammation with an implication of the microglia [21,22]. Thus, microorganisms that composed the microbiota seem to be the recruiters for the bidirectional communication between the gut and the nervous system (Figure 1), and to modulate brain development, function, and behavior [23]. It has even been suggested that gut microbiota control our appetite and the preference for foods that serve them as a substrate [24]. 

The immune system also connects the brain and the microbiota [12]. Concerning the connections to the immune response and its relationship with the microbiota, we can classify the relationship in three terms: (1) when the brain–blood barrier (BBB) is damaged and allows molecules produced, direct or indirectly, by the microbiota, as LPS or the inflammatory interleukin IL-6, to cross the barrier producing neuroinflammation (“leaky gut” theory); (2) the microglia involvement in neurodegeneration and neuroinflammation; and (3) the immune cells, as maturation and function of both, T and B-cells (and also its Ig production) are under the microbiota control [25]. As described by Liang et al. all the mentioned factors can have an impact on AN [26].

Although the pathophysiological mechanisms behind the AN are still to be elucidated, a neuropeptide and neuroendocrine dysregulation is usually found in eating disorders [27]. In AN there is a dysregulation of the endocrine system including the hypothalamic–pituitary axis hormones, adipokines, and appetite-regulating hormones [28]. Emerging data point to a dysregulation of serotonin pathways in cortical and limbic structures that may be related to anxiety, behavioral inhibition, and body image distortions that happen in patients diagnosed of AN [29]. The anxiety and stress associated with AN disorder can be relevant factors altering the microbiota, since prolonged psychological stress induce changes in gut microbiota and in their associated metabolites [30]. It has been demonstrated an inverse relationship between stress and the abundance of *Lactobacillus* [31] and a positive relationship between *Campylobacter* and stress [32]. It seems that stress-induced dysbiosis is the key between chronic psychological stressors and systemic inflammation in humans [33].

## 3. Dysbiosis in Anorexia Nervosa Patients

Gut microbiota may play a vital role in eating disorders. Diet is the most influential external factor for gut microbiota composition and the starvation and emotional imbalance that occurs in AN can induce several changes in gut microbiota. Data available on microbiota in AN population show marked differences in the AN patient’s microbiota when compared with the one of the normal weight individuals. The gut microbiota of patients with anorexia nervosa has lower microbial diversity, affecting all taxonomic levels [34], and there is an overgrowth of some families such as *Enterobacteriaceae* and the archaeon *Methanobrevibacter smithii* (*M. smithii*) [35,36,37]. In addition, it has been observed that there are differences in the microbiota between restrictive anorexia and compulsive anorexia [38]. Some of the taxa underrepresented in the gut microbiota of AN patients are short chain fatty acid (SCFA) producers and it has been observed a negative correlation between the butyrate production and the anxiety levels [36]. Diversity and composition of gut microbiota have been also associated with the depression, anxiety and eating disorder psychopathology symptoms [30]. Current research supports that a decrease of *Firmicutes* with respect to the *Bacteroidetes* together with an increase of the archaea *M. smithii* and *Proteobacteria* could be the microbiota signature associated to AN [38,39]. This archaeon plays an important role removing hydrogen excess from bacterial fermentation in the gut. Its presence seems to be related to the optimization of energy use in the hypocaloric diets followed by AN patients. The presence of *M. smithii* has been correlated with 22 bacterial taxa, mainly clostridiales, establishing a niche of bacterial partners with a syntrophic relationship [40]. Borgo et al. reported increases levels of *M. smithii*, a decrease of *Roseburia*, *Ruminococcus*, and *Clostridium*, followed by a reduction in the butyrate production, possibly due to the decrease of *Roseburia* and *Clostridium*, butyrate producers. They also observed an increase of *Enterobacteriaceae*, which is normally associated with intestinal inflammation and can produce the bacterial peptide caseinolytic protease b (ClpB), an antigen mimeting the a-MSH that suppress appetite through the activation of anorexigenic neurons [36]. Female patients with eating disorders had elevated levels of ClpB that positively correlated with the eating disorder inventory 2 score (EDI-2) [41]. This work would indicate that bacterial peptides could have a role in eating disorders. On the other hand, the secretion of antibacterial peptides such as defensins or immunoglobulin A (IgA), which play an important role in the health of the host by maintaining symbiosis and avoiding colonization by pathogens, could be altered by the AN intestinal dysbiosis [42]. Nowadays, there is no work in the literature that addresses changes in the secretion of antibacterial peptides with the intestinal dysbiosis that occurs in patients with AN.

Diet shapes the composition of gut microbiota, defining which microorganisms colonize, growth, persist, or become extinguished, and therefore also influences the health of the host [43,44]. The lack of food intake in AN could significantly modify the host-gut microbiota. In fact, the microbiota of undernourished children has an immature profile and could be implied in their stunted growth [45,46]. The lack of food can affect the *Lactobacillus* population that usually live in nutrient rich environments and that enter the stationary phase of growth when there are not enough nutrients [47]. There are several populations of bacteria (*Bifidobacterium*, *Bacteroides* and *Verrucomicrobia*) that feed on diet non-digestible carbohydrates and that in the absence of them can use the host-derived glycans, in particular, the mucins that constitute the protective layer of the intestine [48,49,50]. Due to their proximity to the immune system, mucins-degrading bacteria are in a prime location to influence the immune host response and could explain the increase risk of opportunistic infections that suffer AN patients [51]. *Bacteroides* synthesize their membrane exopolysaccharides (EPS) from host glycans. These EPS make them more resistant and in turn reinforce the host’s immune system [49]. If in periods of starvation, such as AN, there is a co-association or competition and exclusion of species that feed on mucins (as *Bifidobacterium*, *Bacteroides* and *Verrucomicrobia*) is something that is still unknown today. Mack et al. [38] have found elevated levels of *Verrucomicrobia* phylum and a lower abundance of the *Bacteroides* genus in patients with AN. When patients gain weight, bacterial diversity increases, but many of the disturbances that have arisen, are maintained [38,52]. (Figure 1; Table 1).

## 4. The Role of Lifestyle in Microbiota Patterns

The etiology of AN is complex. The microbiota dynamics depends on complex interactions between genetics and environmental factors and the microbiome is needed to keep a healthy state. Data suggest that environmental and psychological factors can fire the expression of the associated genetic risk to cause the eating disorder [53]. Concerning the environment, the impact of dietary and non-dietary lifestyle factors on the gut microbiota is well known but not many data are available for AN patients. It seems to be a direct consequence of the disordered feeding behaviors such as vomiting or laxative practices as a part of an unhealthy lifestyle. The discussion about if some cases of AN could be considered a consequence of an inappropriate lifestyle or feeding behaviors is always in the air, for example, the study of Pro-Ana movements, groups of people discussing about anorexia in the social media define the maintenance of certain lifestyle patterns that keep the obsessive idea of getting a very low weight [54]. Indeed, it is common for patients with AN to avoid carbohydrates in the diet and increase the consumption of protein of animal origin. By obtaining a smaller amount of fiber, a smaller amount of short-chain fatty acids is formed, and the production of branched-chain fatty acids increases changing the microbiota composition. This change in the type of fatty acids may be relevant in eating disorders for metabolic dysfunctions and in insulin resistance [55].

Individuals with AN commonly present a comorbid anxiety disorder [56]. Animal studies in *anx^−^/anx^+^* mice have generated new theories that make a point for this comorbidity in inflammation. Moreover, some microbiota patterns in inflammatory situations are related to the hypothalamus–pituitary–adrenal (HPA) axis response to stressors [13]. Some stressors may promote microbial translocation boosting gut inflammation which eventually has been considered the link between microbiota changes in AN and its onset [57]. In fact, although, the analysis of many inflammation biomarkers in blood reported no changes in patients with severe AN symptoms [58], the commensal microbiota related to inflammation suffers changes in AN patients [55]. In addition, diets that are used for recovery, based on animal products, could lead to the development of microbiota with an inflammatory profile [7].

The microbiota patterns in AN, and its changes from the onset to the first stages, could probably give us information about the individual cases and the implications of the different actors in the eating disorder development. It has been reported that early life perturbations can impact neurodevelopment and being responsible of mental problems suffered later in life as both seems to evolve in parallel. This is a point where a lifestyle-based therapeutic intervention should to be consider to combat future brain disorders [59]. 

## 5. Managing Microbiota in Anorexia Nervosa

There are two ways to manipulate microbiota: By manipulating content after the administration of pro/prebiotics or by direct transfer of gut microbiota from another organism. The treatment for eating disorders consists of psychological/psychiatric therapy and nutritional rehabilitation tailored to the patient. However, the biological part of the AN pattern is not usually considered for the treatment, as there are not biological biomarkers that can be used as predictors for the onset and development of AN. Up to date, it is still not clear if the gut microbiota plays a role in the onset and evolution of the disorder but the therapeutic potential of a diet rich in probiotics and prebiotics or the complementation with some probiotic strain look promising [60].

The International Scientific Association for Probiotics and Prebiotics (ISAPP) defines probiotics as living microorganisms that, when administered in adequate quantities, confer benefits for host health [61]. The ISAPP panel supports the general benefits of probiotics for gut microbiota, confirming that they help to have a healthy digestive tract. It mentions, as well, the growing research concerning its use in improving the reproductive tract, oral cavity, lungs, skin, and the microbiota–gut–brain axis [61]. Some probiotics were reported as supplements that improve emotions [62] and certain *Lactobacillus* strains improved behavioral abnormalities [63]. Moreover, the use of the probiotic strain *Bacteroides fragilis* has been proposed in order to correct gastrointestinal function, and it seems to be implicated in the serotonin production restoration [64]. Some other examples of strains studied for the general health benefits mentioned above are *Bifidobacteria* (*B. adolescentis, B. animalis, B. bifidum, B. brevis*, and *B. longum*) and *Lactobacillus* (*L. acidophilus, L. casei, L. fermentum, L. gasseri, L. johnsonii, L. paracasei, L. plantarum, L. rhamnosus*, and *L. salivarius*). Recently, it has been suggested that the supplementation with the probiotic strain *Lactobacillus plantarum* P8 alleviates stress and anxiety patterns that could be related to AN as we stated above [65].

These treatments must be in consonance with the nutritional rehabilitation, with the aim to restore physiological functions by reversing malnutrition. It has to be underlined that patients can respond to them in an unusual way [66]. The use of probiotics strain in AN must be managed carefully due to the risk of bacterial infections. Although these infections are not common, opportunistic microorganisms can take the advantage of infecting immunosuppressed patients like those with malnutrition habits [51]. There are some data on gut microbiota patients with AN suggesting that refeeding increases gut microbial diversity [30]. For this reason, the characterization of the bacterial population through the alpha and beta diversities of patients should be also considered. 

Furthermore, the metabolites from prebiotic fermentation, especially those of SCFA as acetate, propionate, butyrate, and lactate are reported to be beneficial for health, [67]. Concerning the prebiotics, studies in mice show that the use of fructans as prebiotics, reduces obesity, diabetes, hepatic steatosis, inflammation, and insulin resistance and, at the same time, promotes the secretion of peptide YY and GLP1 (glucagon-like peptide-1) [66]. A debate has been opened regarding fiber classification as a prebiotic: not all fibers can be classified as such and the only ones that fit in this category are the fructo-oligosaccharides (FOS) and the galacto-oligosacáridos (GOS) [68]. Inulin is the most well-known type of FOS. It has been shown that inulin can inhibit the intestine colonization from pathogens, providing a protective effect against acute or chronic gut disturbances [67]. 

Considering other way to manipulate microbiota, the transfer of microbiota between organisms can be done using fecal microbiota transplantation (FMT). For mental disorders, transplantation of fecal samples from patients with depression into germ-free mice result in depressive patterns [69]. Upcoming studies will tell us if this is the approximation to restore the microbiota diversity in patients with an impaired microbiota.

Then, taking into consideration the information managed, a protocol including the microbiota study and a possible intervention to replace the damaged microbiota in dysbiosis could be considered. Some of the interesting parameters for studying the microbiota are summarized in three aspects that may be included in the protocol: (1) check nutritional status, dietary patterns and fiber consumption; (2) microbiota analysis focusing in the study of bacterial diversity, the estimation of the *Firmicutes*-*Bacteroidetes* ratio and the total count of *Proteobacteria* and *M. smithii*, through metagenomic studies; and (3) in case of inadequate feeding habits, correct the diet, increasing the fiber intake and consider the supplementation with probiotics strains or food rich in probiotics.

## 6. Conclusions

The intestinal microbiota changes depend on the feeding habits. *M. smithii*, have been found incremented in patients with AN and could be considered a marker for the disease onset and progression. Also, the loss of diversity and changes in the bacterial community composition due to a dysbiosis and the presence of inadequate feeding habits could be a signature to start with the microbiota handling in patients with AN. The intervention could include the restoration of impaired microbiota with different tools as pro/prebiotics intake or a FMT.

Thus, considering the available data, and, in the absence of good biomarkers for the study of AN, microbiota could be a good point of intervention in the management of AN patients. This protocol must be in consonance with a controlled refeeding, increasing the levels of dietary fiber and the use of certain pro/prebiotics that could provide us one step ahead in the knowledge of the complex scenario found in AN patients.

## Figures and Tables

**Table 1:** Differences of gut microbiota when comparing AN patients and controls.

Year Of Publication	Results in AN Patients	Reference
2009	- No changes in *Bacteroidetes*, *Firmicutes* or *Lactobacillus* - Increase in *Methanobrevibacter smithii*	[35]
2013	- Higher levels of *Escherichia coli* - Lower levels of *Lactobacillus reuteri* - Increase in *Methanobrevibacter smithii*	[37]
2015	- Lower alpha diversity - Greater levels of *Bacilli* and unspecified genera in *Coriobacteriales* - Reduced levels of *Clostridia*, *Anaerostipes* and *Faecalibacterium*	[30]
	- Lower amounts of total bacteria and decrease in *Streptococcus* genus, *Clostridium coccoides* group, *Clostridium leptum* subgroup, *Bacteroides fragilis* group, and *Lactobacillus plantarum*	[15]
2016	- No differences in diversity and richness - Decrease in *Bacteroidetes* and increase in *Firmicutes* - Higher levels of mucin-degraders (*Verrucomicrobia, Bifidobacteria, Anaerotruncus*) and members of Clostridium clusters I, XI and XVIII - Reduced abundance of butyrate-producing bacteria (*Roseburia* spp. and *Gemminger* spp.)	[38]
2017	- Lower alpha diversity - Increase in *Coriobacteriaceae*	[34]
	- Decrease in *Firmicutes* - Increase of *Enterobacteriaceae* and *Methanobrevibacter smithii* - Decrease in *Roseburia*, *Ruminococcus* and *Clostridium*	[36]
2018	- Lower alpha diversity - Decrease in: *Eubacterium, Roseburia, Anaerostipes* and *Peptostreptococcaceae* - Increase in: *Turicibacter, Anaerotruncus, Salmonella* and *Klebsiella*	[51]
